# Supplementary material for: Identification of photoperiod-induced specific miRNAs in the adrenal glands of Sunite sheep (Ovis aries)
Source: Front Vet Sci. 2022 Jul 22;9:888207. doi: 10.3389/fvets.2022.888207 (PMC9354845; doi:10.3389/fvets.2022.888207)
Supplement: Supplementary file 1 [file Data_Sheet_1.ZIP › Supplementary file/miRDeep2 file.zip!/miRDeep2 file/Know_Structure/SP42A3/oar-mir-125b.pdf]

miRBase precursor : oar-mir-125b
Total read count : 165180
oar-miR-125b read count : 164613
remaining reads : 567

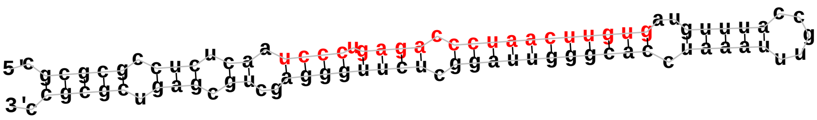

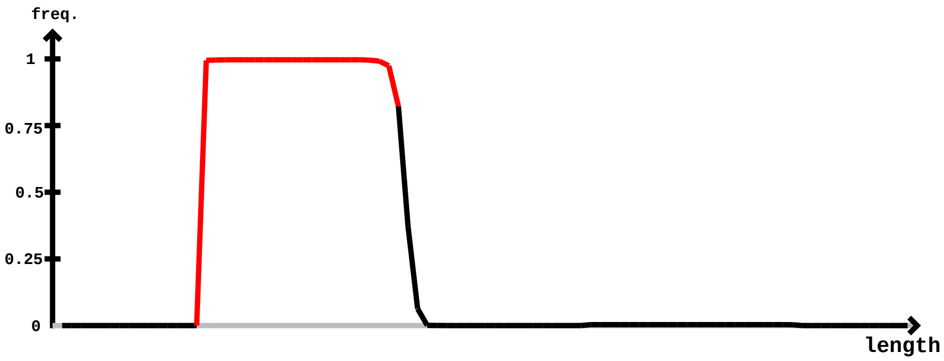


```
                 oar-miR-125b
5'- cgcgcgccucucaaucccugagacccuaacuugugauguuuaccguuuaaaauccacggguuaggcucuugggagcugcgagucgcgcc   -3'   exp
    .(((((.(((.((.((((.((((.((((((((((..(((((.....))))).))))))))))).)))))))).)).))).))))).   reads   mm   sample
    .............Gucccugagacccuaacuugug......................................................   1   1   seq
    .............aucccugagacccuaacuuguga.....................................................   1   0   seq
    ..............ucccAgagacccuaacu..........................................................   2   1   seq
    ..............ucccugagUcccuaacu..........................................................   1   1   seq
    ..............ucccugagacccuaacC..........................................................   27   1   seq
    ..............Acccugagacccuaacu..........................................................   1   1   seq
    ..............ucccugagacccuaacu..........................................................   198   0   seq
    ..............ucccugagacccuaacCu.........................................................   2   1   seq
    ..............ucccugagacccuaacuu.........................................................   559   0   seq
    ..............ucccugagacccuaacuug........................................................   2908   0   seq
    ..............ucccugagacccuaacuugu.......................................................   25057   0   seq
    ..............ucccugagacccuaacuugug......................................................   74292   0   seq
    ..............ucccugagacccuaacuuguga.....................................................   50829   0   seq
    ..............ucccugagacccuaacuugugau....................................................   7376   0   seq
    ..............ucccugagacccuaacuugugaG....................................................   2896   1   seq
    ..............ucccugagacccuaacuugugaug...................................................   49   0   seq
    ..............ucccugagacccuaacuugugaGg...................................................   24   1   seq
    ..............ucccugagacccuaacuugugaugu..................................................   49   0   seq
    ...............cccugagacccuaacuug........................................................   1   0   seq
    ...............cccugagacccuaacuugu.......................................................   9   0   seq
    ...............cccugagacccuaacuugug......................................................   17   0   seq
    ...............cccugagacccuaacuuguga.....................................................   156   0   seq
    ...............cccugagacccuaacuugugaG....................................................   1   1   seq
    ...............cccugagacccuaacuugugau....................................................   7   0   seq
    ................ccugagacccuaacuug........................................................   4   0   seq
    ................ccugagacccuaacuugu.......................................................   24   0   seq
    ................ccugagacccuaacuugug......................................................   59   0   seq
    ................ccugagacccuaacuuguga.....................................................   49   0   seq
    ................ccugagacccuaacuugugau....................................................   8   0   seq
    ................ccugagacccuaacuugugaG....................................................   2   1   seq
    .................cugagacccuaacuugu.......................................................   1   0   seq
    .................cugagacccuaacuuguga.....................................................   2   0   seq
    .................cugagacccuaacuugugaGgu..................................................   1   1   seq
    ....................................................uccacggguuaggcucuuggga...............   1   0   seq
    ......................................................cacggguuaggcucuugggagcu............   1   0   seq
```

oar-miR-125b

```
cgcgcgccucucaaucccugagacccuaacuugugauguuuaccguuuaaauccacggguuaggcucuugggagcugcgagucgcgcc
......................................................acggguuaggcucuuggg................    1          0          seq
......................................................acggguuaggcucuugggag..............    4          0          seq
......................................................acggguuaggcucuugggagc.............    54         0          seq
......................................................acggguuaggcucuugggagcu............    504        0          seq
......................................................acggguuaggcucuugggagcug...........    2          0          seq
```
